# State-Level Health Care Expenditures Associated With Disability

**DOI:** 10.1093/geroni/igab046.600

**Published:** 2021-12-17

**Authors:** Wayne Anderson, Olga Khavjou, Amanda Honeycutt, Laurel Bates, NaTasha Hollis, Scott Grosse, Hilda Razzaghi

**Affiliations:** 1 RTI International, Research Triangle Park, North Carolina, United States; 2 Centers for Disease Control and Prevention (CDC), Atlanta, Georgia, United States

## Abstract

This study updated prior (2003) state-level estimates of disability-associated health care expenditures (DAHE). We combined 2013-2015 data from three national data sets to estimate using multivariate regression all state-level DAHE for US adults in total, by payer, and per adult and per (adult) person with disability (PWD). In 2015, DAHE were $868 billion nationally (State range, $1.4 billion to $102.8 billion) accounting for 36% of total health care expenditures (range, 29%-41%). From over a decade ago, total DAHE increased by 65% (range, 35%-125%). DAHE per PWD was $17,431 (range $12,603 to $27,839). From over a decade ago, per-PWD DAHE increased by 13% (range, –20% to 61%). In 2015, Medicare DAHE per PWD ranged from $10,067 to $18,768. Medicaid DAHE per PWD ranged from $9,825 to $43,365. DAHE are substantial and vary by state and payer. Stakeholders can use these results to develop public health programs to support people with disabilities.

